# Nephrectomy Type Was Not Associated with a Subsequent Risk of Coronary Heart Disease: A Population-Based Study

**DOI:** 10.1371/journal.pone.0163253

**Published:** 2016-09-16

**Authors:** Shiu-Dong Chung, Chao-Yuan Huang, Sheng-Tang Wu, Herng-Ching Lin, Chung-Chien Huang, Li-Ting Kao

**Affiliations:** 1 Division of Urology, Department of Surgery, Far Eastern Memorial Hospital, Banciao, Taiwan; 2 Graduate Program in Biomedical Informatics, College of Informatics, Yuan-Ze University, Chung-Li, Taiwan; 3 Sleep Research Center, Taipei Medical University Hospital, Taipei, Taiwan; 4 School of Public Health, Taipei Medical University, Taipei, Taiwan; 5 Department of Urology, National Taiwan University Hospital, College of Medicine National Taiwan University, Taipei, Taiwan; 6 Division of Urology, Department of Surgery, Tri-Service General Hospital and National Defense Medical Center, Taipei, Taiwan; 7 School of Health Care Administration, Taipei Medical University, Taipei, Taiwan; 8 Graduate Institute of Life Science, National Defense Medical Center, Taipei, Taiwan; National Health Research Institutes, TAIWAN

## Abstract

Previous studies investigated the impacts of a partial nephrectomy (PN) or radical nephrectomy (RN) on cardiovascular events and death. However, the association between the type of nephrectomy (PN vs. RN) and cardiovascular disease is still equivocal. This retrospective cohort study aimed to compare the risk of coronary heart disease (CHD) between patients who underwent a PN and those who underwent an RN. We used data from the Taiwan Longitudinal Health Insurance Database 2005. In total, 60 patients who underwent a PN and 545 patients who underwent an RN were included. Each patient was tracked for 1-, 2-, 3-, and 5-year periods to identify those who were subsequently diagnosed with CHD. Cox proportional hazard regression analyses were used to calculate hazard ratios (HRs) for CHD during 1-, 2-, 3-, and 5-year follow-up periods between these two cohorts. For the 1-year follow-up period, the adjusted HR was 0.39 (95% CI: 0.05~2.90, *p* = 0.355) for patients who underwent a PN compared to those who underwent an RN. Additionally, the adjusted HRs of CHD in patients who underwent a PN for 2-, 3- and 5-year follow-up periods were 1.40 (95% CI: 0.62~3.16, *p* = 0.417), 1.09 (95% CI: 0.52~2.31, p = 0.814), and 1.02 (95% CI: 0.48~2.18, *p* = 0.961), respectively, compared to those who underwent an RN. We concluded that there was no significant difference in the risk of CHD between patients who underwent a PN and those who underwent an RN.

## Introduction

Radical nephrectomy (RN) is recognized as a gold standard treatment for localized renal cell carcinomas [1, 2]. This surgery would remove the entire kidney and all the contents within the renal fascia [3]. Nevertheless, in comparison to an RN, the partial nephrectomy (PN) could preserve the renal parenchyma uninvolved by the tumor at the time of surgery [4]. Therefore, although an RN has been the common treatment of small renal tumors in past decades, a PN is now the preferred treatment for patients with small renal masses [5, 6]. To date, increasing evidences showed that PN and RN have similar outcomes for T1 renal tumors, including the rate of cancer-specific mortality and local or distant recurrence [7]. Moreover, many studies indicated that this surgery may protect long-term renal function and decrease the risk of subsequent chronic kidney disease (CKD) since a PN can preserve patients' renal parenchyma [8–12].

Exacerbation of renal function and CKD are both risk factors for the development of cardiovascular diseases [13, 14]. Additionally, coronary heart disease (CHD) is a primary cause of death in general populations and in patients with CKD [15, 16]. Accordingly, it is plausible that PN might decrease the incidence of CHD because PN can preserve renal parenchyma and protect renal function [8–16]. However, to date, most literatures only attempted to investigate the association between nephrectomy types and oncologic or overall survival [12, 17–23]. Only a few studies have attempted to investigate differences in cardiovascular mortality and morbidity between patients who underwent a PN and those who underwent an RN [24–27]. For instance, one study in the United States reported no difference in postoperative cardiovascular morbidity rates between a PN and RN [24]. Another study also found that the type of nephrectomy (PN vs. RN) was not an independent predictor of cardiac-specific deaths [25].

Conversely, two studies conducted in the United States both showed that an RN was significantly associated with a higher cardiovascular mortality than was a PN [26, 27]. Therefore, the associations between nephrectomy type and cardiovascular morbidity and mortality are still inconsistent. Additionally, all relevant studies were conducted in western countries. To our best knowledge, no study to date has directly examined the relationship between the nephrectomy type and the risk of cardiovascular diseases in an Asian country. Therefore, this study aimed to explore the relationship between nephrectomy type and the subsequent risk of CHD using a large population-based dataset in Taiwan.

## Methods

### Database

This study used data from the Taiwan Longitudinal Health Insurance Database 2005 (LHID2005). The LHID2005 involves original claims data and registration files for 1 million individuals randomly selected from all enrollees in the Taiwan National Health Insurance (NHI) program in 2005 (*n* = 25.68 million). The NHI program in Taiwan is a single-payer system and approximately 99.9% of Taiwanese population were registered in this system. Additionally, this NHI program was initiated in 1995 and provides accessible and affordable medical services for all citizens in Taiwan. The LHID2005 allows researchers to trace all medical records (including physician diagnoses, medications, treatments, and surgeries, etc.) of these 1 million enrollees since the beginning of Taiwan's NHI program. This population-based database is released to researchers in Taiwan for academic purposes by the Taiwan National Health Research Institutes (http://nhird.nhri.org.tw/en/index.html). It consists of de-identified secondary data and a number of studies have been published in international peer-reviewed journals to date.

### Study Sample

This study was a retrospective cohort study and included a study cohort and a comparison cohort. We selected the study cohort by first identifying 84 patients who underwent a PN (ICD-9-CM procedure code 554) in January 2001 to December 2010. The date of the PN was defined as the index date for the study cohort, and the procedure codes were made by a certified urologist. We then excluded patients who had been diagnosed with CHD (ICD-9-CM codes 410~414 or 429.2) (*n* = 24) prior to the index date. Finally, 60 patients who underwent a PN were included in the study cohort.

For the comparison cohort, we initially defined 782 patients who underwent an RN between January 2001 and December 2010 based on the ICD-9-CM procedure code 555. The date of the RN was identified as the index date. We then excluded 237 patients who had a history of CHD prior to their index date. Ultimately, 545 patients who underwent an RN were identified as the comparison cohort.

### Outcome Measures

In this study, each patient (*n* = 605) was individually tracked for a 1-, 2-, 3-, and 5-year period to define those who received a diagnosis of CHD (ICD-9-CM codes 410~414 or 429.2) during the period from January 2001 to December 2013 after the index date.

### Statistical Analysis

All analyses in this study were conducted with the SAS system (SAS System for Windows, vers. 9.2, SAS Institute). Chi-squared tests were performed to compare differences in sex, monthly income, geographic location, urbanization level, and patients' comorbidities (including hypertension, diabetes mellitus, hyperlipidemia, and chronic renal failure) between patients who underwent a PN and those who underwent an RN. These medical comorbidities were only included if they were diagnosed prior to the index date. Student's *t*-test was conducted to investigate differences in age between patients who underwent a PN and those who underwent an RN.

Thereafter, Cox proportional hazard regression analyses were used to calculate hazard ratios (HRs) for CHD during 1-, 2-, 3-, and 5-year follow-up periods between these two cohorts. We censored patients who died during the follow-up period. Additionally, in order to avoid the potential effect of the cardiovascular risk factors, renal function, and patients’ demographics on the association between a PN and CHD, we estimated the risk of CHD by taking hypertension, diabetes, hyperlipidemia, chronic renal failure, patients’ age, sex, geographical location, monthly income, urbanization level into consideration in the regression models. This study showed HRs along with 95% confidence intervals (CIs). Statistical significance was set at a conventional two-sided *p* value of <0.05.

## Results

This study included 60 patients who underwent a PN as the study cohort and 545 patients who underwent an RN as the comparison cohort. Of the 605 patients, the mean age was 56.7 years with a standard deviation of 16.1 years. Mean ages for the study and comparison cohorts were 53.6 and 57.0 years, respectively (*p* = 0.202). The demographic characteristics and comorbidities of the sampled patients are shown in Table 1. Patients who underwent a PN had a significantly lower prevalence of chronic renal failure than those who underwent an RN (5.0% vs. 16.7%, *p* = 0.018). However, there was no significant difference in monthly income, geographic region, urbanization level, or some comorbidities, including hypertension, diabetes, and hyperlipidemia, between the study and comparison cohorts.

**Table 1:** Demographic characteristics of patients who underwent a partial nephrectomy (PN) and those who underwent a radical nephrectomy (RN) (*N* = 605).

Variable	Patients who underwent a PN		Patients who underwent an RN		*p* value
	*n =* 60		*n =* 545		
	Total no.	Column %	Total no.	Column %	
Age (years)	53.6±14.2		57.0±16.3		0.202
Sex					0.596
Male	28	46.7	274	50.3	
Female	32	53.3	271	49.7	
Monthly income					0.122
≤NT$15,840	26	43.3	267	49.0	
NT$15,841~25,000	18	30.0	190	34.9	
≥NT$25,001	16	26.7	88	16.2	
Geographical region					0.277
Northern	30	50.0	213	39.1	
Central	9	15.0	133	24.4	
Southern	20	33.3	193	35.4	
Eastern	1	1.7	6	1.1	
Urbanization level					0.215
1 (most urbanized)	20	33	161	29.5	
2	21	35	151	27.7	
3	11	18	83	15.2	
4	4	6.7	69	12.7	
5 (least urbanized)	4	6.7	81	14.9	
Comorbidities					
Hypertension	20	33.3	181	33.2	0.985
Diabetes mellitus	14	23.3	93	17.1	0.227
Hyperlipidemia	10	16.7	86	15.8	0.858
Chronic renal failure	3	5.0	91	16.7	0.018

The average exchange rate in 2011 was US$1.00≈New Taiwan (NT)$30.

Table 2 presents the incidence rates for CHD among sampled patients. Incidence rates of CHD per 100 person-years within the 1-year follow-up period were 1.68 (95% CI: 0.43~9.38) and 5.14 (95% CI: 3.36~7.53) for patients who underwent a PN and those who underwent an RN, respectively. Incidence rates of CHD per 100 person-years within the 2-year follow-up period were 5.96 (95% CI: 2.40~12.29) for patients who underwent a PN and 5.64 (95% CI: 4.20~7.41) for those who underwent an RN. Moreover, incidence rates of CHD per 100 person-years within the 3-year follow-up period were 4.59 (95% CI: 1.98~9.05) and 5.45 (95% CI: 4.25~6.89), respectively, for patients who underwent a PN and those who underwent an RN. Additionally, the incidence rates of CHD per 100 person-years within the 5-year follow-up period were 3.98 (95% CI: 1.91~7.31) and 5.00 (95% CI: 4.06~6.12) for patients who underwent a PN and those who underwent an RN, respectively.

**Table 2:** Prevalences, hazard ratios (HRs), and 95% confidence intervals (CIs) for coronary heart disease among the sampled patients.

Presence of coronary heart disease	Patients who underwent a partial nephrectomy (*n =* 60)	Patients who underwent a radical nephrectomy (*n =* 545)
One-year follow-up period		
Incidence rate per 100 person-years (95% CI)	1.68 (0.43~9.38)	5.14 (3.36~7.53)
HR (95% CI)	0.34 (0.05~2.53)	1.00
Adjusted HR ^a^ (95% CI)	0.39 (0.05~2.90)	1.00
Two-year follow-up period		
Incidence rate per 100 person-years (95% CI)	5.96 (2.40~12.29)	5.64 (4.20~7.41)
HR (95% CI)	1.23 (0.56~2.71)	1.00
Adjusted HR ^a^ (95% CI)	1.40 (0.62~3.16)	1.00
Three-year follow-up period		
Incidence rate per 100 person-years (95% CI)	4.59 (1.98~9.05)	5.45 (4.25~6.89)
HR (95% CI)	1.04 (0.50~2.15)	1.00
Adjusted HR ^a^ (95% CI)	1.09 (0.52~2.31)	1.00
Five-year follow-up period		
Incidence rate per 100 person-years (95% CI)	3.98 (1.91~7.31)	5.00 (4.06~6.12)
HR (95% CI)	0.94 (0.46–1.91)	1.00
Adjusted HR ^a^ (95% CI)	1.02 (0.48~2.18)	1.00

Notes: Using Cox proportional regressions with cases censored if patients died during the follow-up period.

^a^ Adjustments were made for patients’ age, sex, geographical location, monthly income, urbanization level, hypertension, diabetes, hyperlipidemia, and chronic renal failure.

The HRs for subsequent CHD in patients who underwent a PN compared to those who underwent an RN are also shown in Table 2. For the 1-year follow-up period, the adjusted HR was 0.39 (95% CI: 0.05~2.90, *p* = 0.355) for patients who underwent a PN compared to those who underwent an RN after adjusting for patients’ age, sex, monthly income, geographical location, urbanization level, and comorbidities. Furthermore, the adjusted HRs of CHD in patients who underwent a PN for 2-, 3- and 5-year follow-up periods were 1.40 (95% CI: 0.62~3.16, *p* = 0.417), 1.09 (95% CI: 0.52~2.31, p = 0.814), and 1.02 (95% CI: 0.48~2.18, *p* = 0.961), respectively, compared to those who underwent an RN.

## Discussion

This retrospective cohort study found that patients who underwent a PN did not have an elevated risk of subsequent CHD for the 1-, 2-, 3- or 5-year follow-up periods compared to those who underwent an RN. According to our best knowledge, no previous study has attempted to explore the association between the nephrectomy type and CHD, although a few studies suggested that preserving renal function might protect patients' cardiovascular system and decrease the occurrence of cardiovascular diseases [13, 14].

To date, most of the literature only indicated that patients who underwent a PN would have lower overall mortality compared to those who underwent an RN [12, 17–22]. Conversely, a randomized trial found that PN seems to be less effective than RN in terms of overall survival [23]. However, very few studies further investigated the relationship of the nephrectomy type with cardiovascular morbidity and mortality to date. Our study found that patients who underwent a PN did not a have higher risk of subsequent CHD compared to those who underwent an RN. Our observation is in light of findings of some prior studies [24–28]. For instance, one retrospective cohort study which used data from the Surveillance, Epidemiology and End Results (SEER) registry in the United States showed that there was no difference in adverse cardiovascular outcomes (including ischemic heart disease-related or congestive heart failure-related hospitalizations or diagnoses) between patients who underwent a PN and those who underwent an RN [24]. In addition, an American study reported that the nephrectomy type was not an independent predictor of cardiac-specific deaths (including deaths from ischemic heart disease, congestive heart disease, ischemic stroke, and peripheral vascular disease) [25]. A randomized study in Europe also observed that there were no significant differences in cardiovascular mortality between a PN and RN [28]. Furthermore, Huang et al. performed a cohort study using the SEER registry and found that patients who underwent an RN did not have significant risks of a first cardiovascular event or cardiovascular death compared to those who underwent a PN [26].

However, results of some previous studies do not parallel our findings. For example, Huang et al. reported that the occurrence of cardiovascular events in patients who underwent an RN was 1.4-fold higher than those who underwent a PN [26]. One study in the United States also showed that patients who underwent an RN had a significantly higher risk of cardiovascular mortality (HR 2.53, 95% CI: 1.51~4.23) compared to those who underwent a PN [27]. Recently, a multi-institutional study in Europe concluded that the risk of subsequent cardiovascular events (including the onset of coronary artery disease, cardiomyopathy, vasculopathy, hypertension, heart failure, dysrhythmias, or cerebrovascular disease) in patients who underwent a PN was 0.57-fold lower compared to those who underwent an RN. In conclusion, based on the above studies, the association between the nephrectomy type and overall cardiovascular outcomes remains unclear. Further large-scale epidemiological studies in other regions or countries are still needed to clarify this association.

The principle strength of this study is the use of the LHID2005 which is a longitudinal population-based dataset in Taiwan. The characteristics of this dataset could increase the statistical power and reduce the potential effects of a selection bias. Nevertheless, this study suffers from some limitations. First, the LHID2005 used in this study contained no information on several potential confounders, including the family history of CHD, body-mass index, dietary habits, cigarette smoking, etc [29]. These are considered to be risk factors for CHD and might further affect the relationship between the nephrectomy type and CHD. Second, there was no laboratory information about patients' renal function, such as the glomerular filtration rate or creatinine clearance rate, in the LHID2005. However, in order to avoid the potential impact of renal function on the relationship between a PN and CHD, we estimated the risk of CHD by taking chronic renal failure into consideration in the regression model. Third, the LHID2005 provide no records about the quality and quantity of preserving renal parenchyma after PN. The amount of renal reservation is considered to be a determinant of post-surgical renal function. Fourth, even though this research was a population-based study, a relatively small sample size of PN cases might potentially affect the association between a PN and subsequent CHD. Finally, most sampled patients in this study were of Chinese ethnicity. Therefore, the ability to generalize the findings to other ethnic groups is still uncertain.

In conclusion, this population-based cohort study showed that there was no significant difference in the risk of subsequent CHD during a 1-, 2-, 3-, or 5-year follow-up period between patients who underwent a PN and those who underwent an RN. We consider that the results of this study have suggestions for patients facing nephrectomy. Additionally, the findings may provide some clinical information for physicians to evaluate the potential risks and benefits of the use of a PN and RN. Nevertheless, further large epidemiologic studies are still required to confirm the relationship between nephrectomy type and subsequent CHD in different ethnicity and countries.
